# Non Edible Oil-Based Epoxy Resins from Jatropha Oil and Their Shape Memory Behaviors

**DOI:** 10.3390/polym13132177

**Published:** 2021-06-30

**Authors:** Lu Lu Taung Mai, Min Min Aung, Sarah Anis Muhamad Saidi, Paik San H’ng, Marwah Rayung, Adila Mohamad Jaafar

**Affiliations:** 1 Higher Education Centre of Excellence (HiCoE), Institute of Tropical Forestry and Forest Products, University Putra Malaysia, Serdang 43400, Malaysia; lulutawngmai@gmail.com (L.L.T.M.); marwahrayung@yahoo.com (M.R.); 2 Department of Chemistry, Universiry of Myitkyina, Kachine State 01011, Myanmar; 3 Department of Chemistry, Faculty of Science, Universiti Putra Malaysia, Serdang 43400, Malaysia; sarah.anisss@gmail.com (S.A.M.S.); adilamj@upm.edu.my (A.M.J.); 4 Chemistry Division, Centre of Foundation Studies for Agricultural Science, University Putra Malaysia, Serdang 43400, Malaysia; 5 Department of Forestry and Environment, Faculty of Forestry, Universiti Putra Malaysia, Serdang 43400, Malaysia

**Keywords:** epoxidized jatropha oil, shape memory polymer, bio-based polymer, jatropha oil

## Abstract

The use of bio-based polymers in place of conventional polymers gives positives effects in the sense of reduction of environmental impacts and the offsetting of petroleum consumption. As such, in this study, jatropha oil was used to prepare epoxidized jatropha oil (EJO) by the epoxidation method. The EJO was used to prepare a shape memory polymer (SMP) by mixing it with the curing agent 4-methylhexahydrophthalic anhydride (MHPA) and a tetraethylammonium bromide (TEAB) catalyst. The resulting bio-based polymer is slightly transparent and brown in color. It has soft and flexible properties resulting from the aliphatic chain in jatropha oil. The functionality of SMP was analyzed by Fourier transform infrared (FTIR) spectroscopy analysis. The thermal behavior of the SMP was measured by thermogravimetric analysis (TGA), and it showed that the samples were thermally stable up to 150 °C. Moreover, the glass transition temperature characteristic was obtained using differential scanning calorimetry (DSC) analysis. The shape memory recovery behavior was investigated. Overall, EJO/MHPA was prepared by a relatively simple method and showed good shape recovery properties.

## 1. Introduction

Shape memory polymers (SMPs) are a class of emerging smart materials that can change shape and “remember” their original shape. They have a sensitive response to external stimuli such as pH, humidity, light, electricity, temperature, and so on [1,2,3]. To date, most of the studies reported on SMPs are stimulated by temperature. In general, SMPs contain two phases: the permanent shape and the temporary shape. The permanent shape can be either covalent or physical cross-links. The temporary shape is set by deformation above a certain transition temperature [4]. They typically rely on vitrification, crystallization, or some other physical interaction. When the polymers are reheated above the transition temperature, the oriented polymer chains are released, resulting in recovery of the permanent shape. SMPs have attracted significant attention due to their inherent advantages like good processability, low cost, and high recovery ability [5]. At present, shape memory polymers have been applied in many fields such as aerospace [6], medicine [7,8], self-finishing smart textiles [9], electronic devices [10], and self-assembling structures [11]. Various types of polymers have been found to have shape memory effects including polyurethane [12,13], trans-polyisoprene [14], styrene-butadiene copolymer [15], and epoxy material [16].

Recently, considerable efforts have been expended to develop bio-based SMPs. Bio-based polymers are gaining attention as they have renewability features couples with their economic and environmental benefits. In essence, there have been substantial studies reported on bio-based epoxy materials for shape memory polymers [17,18,19]. Among all renewable resources, vegetable oils are expected to be an ideal alternative to chemical feedstocks in preparing epoxy materials. Vegetable oil is one of the cheapest and most abundant types of biomass, which is available in large quantities [20]. Vegetable oil is predominantly made up of triglyceride molecules with varying compositions of fatty acids. Some examples of vegetable oils that can be used include palm oil, soybean oil, linseed oil, sunflower oil, castor oil, jatropha oil, and many others [19].

This study focuses on the utilization of jatropha oil as the raw material to prepare epoxy resins for SMP production. Jatropha oil is obtained from the seeds of jatropha curcas tree. Jatropha curcas is a succulent plant that belongs to the Euphorbiaceae family, widely grown in South America, South-West Asia, India, and Africa. The oil content of jatropha seeds was reported at 63%, which is higher than that of palm kernel, linseed, and soybean oil content [21]. Jatropha oil is classified as a non-edible oil due to the presence of toxic components in the structure, such as phorbol ester, curcains, and jatropherol [22], and thus it cannot be used for nutritional purposes without detoxification. By being a non-edible oil, there will be no unnecessary competition with the food-related industries. Other than that, jatropha oil contains high amounts of unsaturation, which is made up mostly by oleic and linoleic acid. The chemical compositions of the oil differ depending on the climate and locality of the jatropha tree. The high degree of unsaturation of the oil offers a wide alternative for chemical modification. In this study, we describe the preparation and characterization of bio-based shape memory materials from epoxidized jatropha oil. Further, the thermal and shape memory properties of the resulting materials are investigated.

## 2. Materials and Methods

### 2.1. Materials

Crude jatropha oil was supplied by Biofuel Bionas Sdn Bhd. Formic acid (98%), hydrogen peroxide (30%), sulphuric acid (95%), methanol (99%), and crystal violet (96%) were supplied by R&M Chemicals, Dundee, UK. Magnesium sulphate (97%), hydrogen bromide (33%), 4-methylhexhydrophthalic anhydride (MHPA) (98%), and tetraethyl ammonium bromide (TEAB) (98%) were obtained from ACROS Organic, Carlsbad, CA, USA. Potassium hydrogen phthalate (99%) was obtained from AJAX Chemicals, Taren Point, Australia, and chlorobenzene (99%) was supplied by Merck, Kenilworth, NJ, USA. All chemicals were used as received.

### 2.2. Preparation of Epoxidized Jatropha Oil

The epoxidized jatropha oil (EJO) was prepared in bulk according to the procedures reported in the literature [23,24]. A mixture of jatropha oil and formic acid was heated to 40 °C under continuous stirring in a water bath. Once the temperature reached 40 °C, hydrogen peroxide was added in a dropwise manner to avoid overheating. Then, the temperature was increased and maintained at 60 °C for 5 h. Oxirane oxygen content (OOC) analysis was conducted every 1 h. After 5 h of stirring, the mixture was cooled down to room temperature. After that, the aqueous layer was discarded, and the produced epoxy was washed with an excess amount of distilled water until neutral pH was obtained. Lastly, a rotary evaporator was used to remove the remaining trace of water in the epoxy to obtain a pure EJO. The sample was kept with MgSO_4_ drying agent and stored in a desiccator.

### 2.3. Preparation of EJO/MHPA

**Figure 1:**
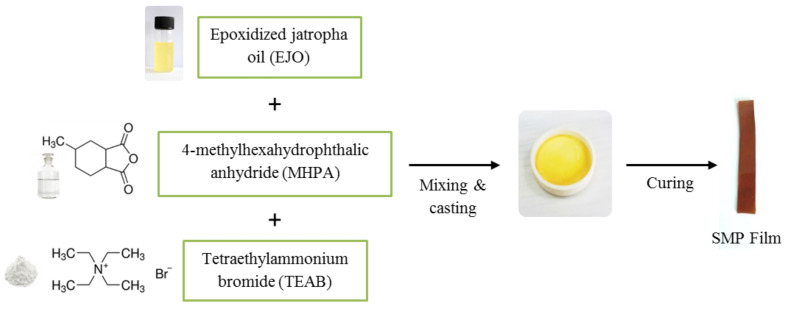
Schematic view of the preparation of jatropha oil-based shape memory polymer (SPM).

The shape memory polymer was prepared by mixing EJO, MHPA, and TEAB, as shown in Figure 1. TEAB was added as the catalyst in the reaction. Various molar ratios of anhydride to epoxy were prepared in this study, which were 0.6, 0.8, 1.0, 1.2, and 1.4. For the molar ratio of 1.0, 8.0 g of EJO, 5.6 g of MHPA, and 0.07 g of TEAB were mixed together [5]. The mixture was degassed under vacuum conditions for 10 min at 80 °C. Then, the mixture was poured into a Teflon mold and was kept in the oven for 22 h at 150 °C. The produced EJO/MPHA polymer was cooled down to room temperature. The same procedure was repeated to prepare SMP by using another molar ratio.

### 2.4. Characterization

#### 2.4.1. Oxirane Oxygen Content

The measurement of oxirane oxygen (OOC) content during the preparation of EJO was carried out according to the ASTM D1652–97 Test Method A Standard [25]. This measurement was used to calculate the conversion of the double bond of the oil to oxirane rings. A total of 0.4 g of EJO was dissolved in 10 mL of chlorobenzene and 3–4 drops of crystal violet were used as the indicator. The mixture was titrated with standardized hydrogen bromide until it reached the endpoint, which was observed by color changes to green. The OOC was calculated based on Equation (1).

(1) $$OOC\;\left(\%\right)=\frac{1.6N\left(V-B\right)}{W}$$

where *N* is the normality of standardized hydrogen bromide solution (N), *V* is the volume of standardized hydrogen bromide used for the EJO sample (mL), *B* is the volume of standardized hydrogen bromide used for the blank sample (mL), and *W* is the weight of the sample (g).

#### 2.4.2. Fourier Transform Infrared Spectroscopy

Fourier transform infrared (FTIR) spectroscopy was used to analyze the presence of different functional groups in the samples. The analysis was conducted by using a SHIDMADZU IR Tracer-100 series spectrophotometer. The IR spectra of samples recorded were in the wavenumber range of 4000 cm^−1^ to 400 cm^−1^ at a scanning rate of 4 cm^−1^.

#### 2.4.3. Nuclear Magnetic Resonance Spectroscopy

The ^1^H-NMR (proton NMR) and ^13^C-NMR (carbon NMR) spectra were recorded using a 500 MHz JEOL Nuclear Magnetic Resonance (Peabody, MA, USA) at room temperature. The samples were dissolved in deuterated chloroform (CDCl_3_). The ^1^H and ^13^C chemical shifts were recorded in ppm and referenced to tetramethylsilane (TMS) at 0.0 ppm.

#### 2.4.4. Thermal Analysis

The thermal properties of the EJO/MHPA polymers with various ratios of anhydride to oxirane were evaluated by using a Perkin Elmer TGA7, Cleveland, OH, USA, thermogravimetric analyzer. This analysis was performed at temperatures ranging from 25 °C to 600 °C at a constant heating rate of 10 °C min^−1^ under a nitrogen atmosphere (flow rate: 50 mL min^−1^). The changes in the weight of the sample as a function of temperature and/or time were measured. Additionally, the glass transition behavior of the SMP was investigated by using a Mettler Toledo, Grelfensee, Switzerland, differential scanning calorimetry (DSC) instrument. The scan was conducted at the rate of 10 °C min^−1^ from −20 °C to 120 °C with a nitrogen gas flow rate of 50° mL min^−1^.

#### 2.4.5. Investigation of the Shape Memory Recovery Behavior of EJO/MHPA Polymer

The shape memory behavior of the EJO/MPHA polymer was investigated by using the cured EJO/MPHA polymer. The samples were cut to produce a linear rectangular shape, and this was taken as their original shape. Then, the sample was heated at 150 °C and cooled down to room temperature, forming a temporary shape. When the temporary shape was heated again at 150 °C, the temporary shape returned back to its original shape.

## 3. Results

### 3.1. Oxirane Oxygen Content

**Figure 2:**
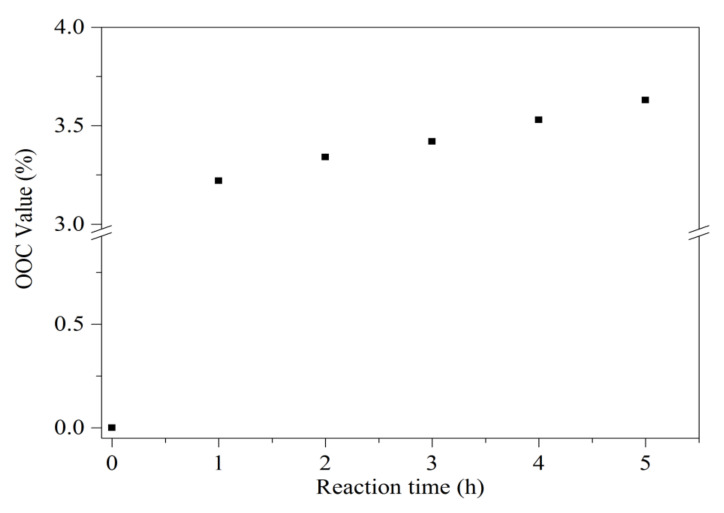
Oxirane oxygen content (OOC) value with time for the epoxidized jatropha oil process.

During the epoxidation process, the double bonds of the unsaturated oil were converted into epoxy rings by using in situ generated performic acid. It was prepared by mixing formic acid and hydrogen peroxide. In this case, formic acid functioned as the oxygen carrier, and hydrogen peroxide acted as the oxygen donor. The reaction was carried out in situ, as the reaction is highly exothermic [26]. The conversion double bonds to epoxy groups were monitored using oxirane oxygen content (OOC) analysis. Figure 2 shows the OOC value against the reaction time pattern. As can be seen in Figure 2, the OOC value gradually increased as the reaction time increased and reached a maximum at 5 h. The produced epoxy jatropha oil had a maximum oxirane oxygen content of 3.63%. Prolonged reaction time will cause the OOC value to drop due to the possibility of the ring-opening reaction to occur. During the epoxidation process, the temperature must be controlled, as a high reaction temperature may intensify the rate of hydrogen peroxide decomposition and may exceed the rate of epoxy formation. As oxygen supplied by the hydrogen peroxide depleted, the epoxy formation ended earlier than anticipated. These unfavorable reactions resulted in lower oxirane oxygen content than the theoretical value. A similar observation of the OOC value trend of EJO was reported by a previous study [27]. Subsequently, the produced EJO in this study was used as the epoxide monomers to prepare EJO/MHPA polymers.

### 3.2. FTIR Analysis

**Figure 3:**
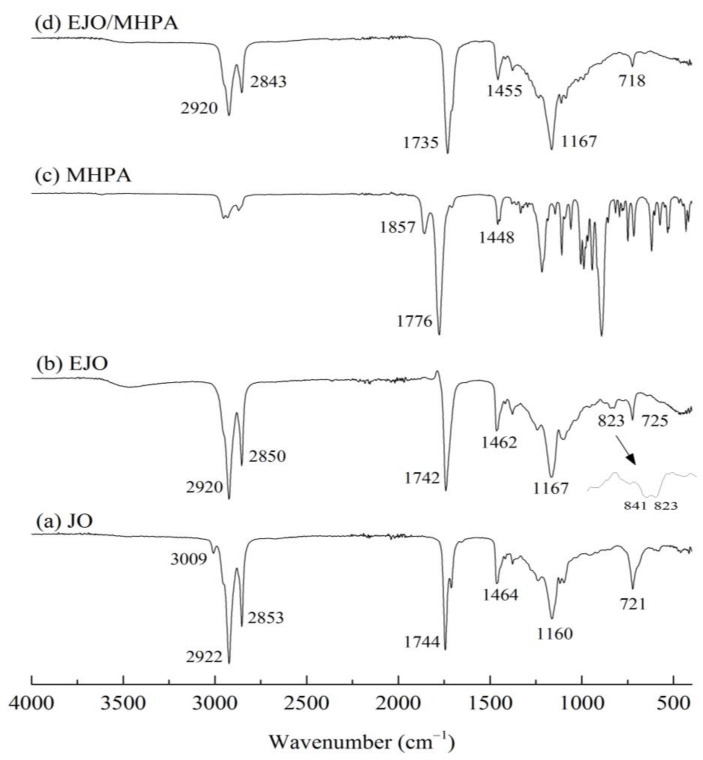
FTIR spectra of (**a**) JO, (**b**) EJO, (**c**) MHPA, and (**d**) EJO/MHPA SMP.

FTIR analysis was conducted to identify chemical changes that occurred during the preparation process to the end product. The epoxidation of double bonds in jatropha oil forming an oxirane group and the ring opening of the oxirane group in the polymerization were observed. Figure 3 shows the FTIR spectra of jatropha oil, EJO, MHPA, and EJO/MHPA polymer. Based on Figure 3a for jatropha oil, the absorption bands at 2853 and 2922 cm^−1^ were attributed to the –CH symmetric and asymmetric stretching, respectively, of –CH_2_ groups [28]. Moreover, the peaks at 721 and 1464 cm^−1^ were ascribed to –CH_2_ rocking and bending vibrations. The intense peak at 1744 cm^−1^ indicated the C=O stretching vibration. The C–O–C stretching vibration of the ester group was located at 1163 cm^−1^. The band at 3009 cm^−1^ was attributed to the C=C double bonds in the oil, which were oleic and linoleic acid [29]. In Figure 3b, the disappearance of the double bond functional group peak at 3009 cm^−1^, which was initially present in the JO spectra, indicates the conversion of the double bond to the epoxy group. The new peaks formed at 823 and 841 cm^−1^ corresponded to the conversion of double bonds into epoxy groups. This is in agreement with similar findings reported by other researchers previously [27,30,31].

**Figure 4:**
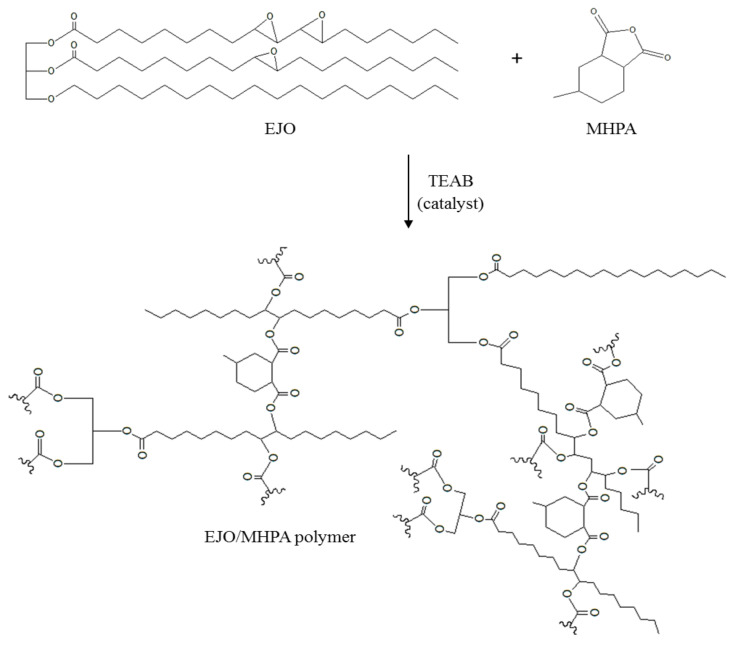
Schematic reaction of EJO and MHPA.

For the MHPA spectrum presented in Figure 3c, peaks of symmetric and asymmetric C=O stretching could be observed at the 1776 and 1857 cm^−1^ regions. Moreover, the spectrum for EJO/MHPA polymer, displayed in Figure 3d, showed a combination of both EJO and MHPA characteristics. A strong adsorption peak of C=O stretching of a carbonyl group present at 1735 cm^−1^ showed the formation of ester bonds between the MHPA and EJO [32]. Furthermore, the epoxy peaks also disappeared for EJO/MHPA polymer, confirming the formation of the bond. It can be deduced that there was a hydrolysis reaction of MHPA [5] and a ring opening of the oxirane group forming the EJO/MHPA polymer. The cross-linked ester was formed, as shown in Figure 4.

**Figure 5:**
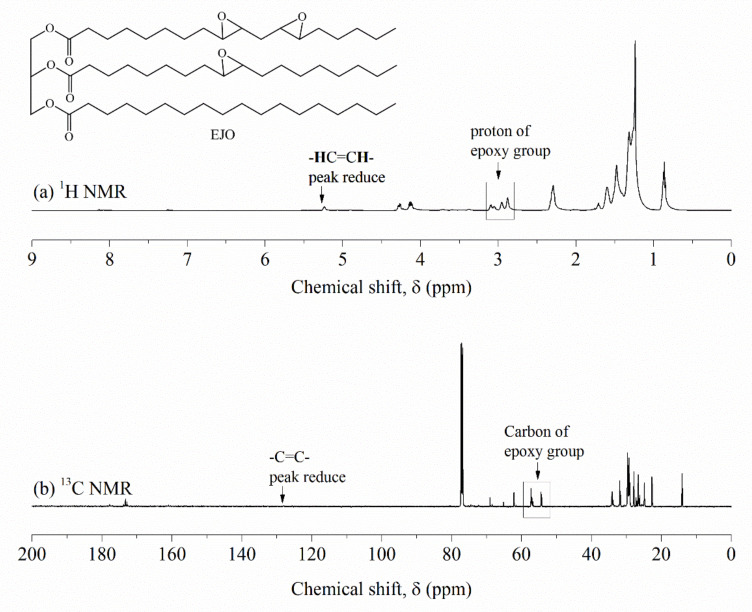
(**a**) ^1^H NMR and (**b**) ^13^C NMR spectra of epoxidized jatropha oil.

The ^1^H NMR and ^13^C NMR spectra of epoxidized jatropha oil are depicted in Figure 5. Upon epoxidation, there was a notable chemical shift in the spectra that could be observed. The unsaturation peak in the jatropha oil almost completely disappeared in the ^1^H NMR spectrum for epoxidized jatropha oil. Moreover, the formation of new peaks in the region of 2.8–3.1 ppm indicated the presence of epoxy group protons [31]. Further, the characteristic unsaturation peaks at 120–130 ppm for ^13^C NMR was absent and accompanied by the appearance of carbon signals at 54–58 ppm showed the presence of epoxy groups. It can be deduced that double bonds in the jatropha oil were successfully converted to epoxy groups. This finding was in agreement with the previous study reported by Sammaiah and co-workers [28].

### 3.3. Thermal Analysis

**Figure 6:**
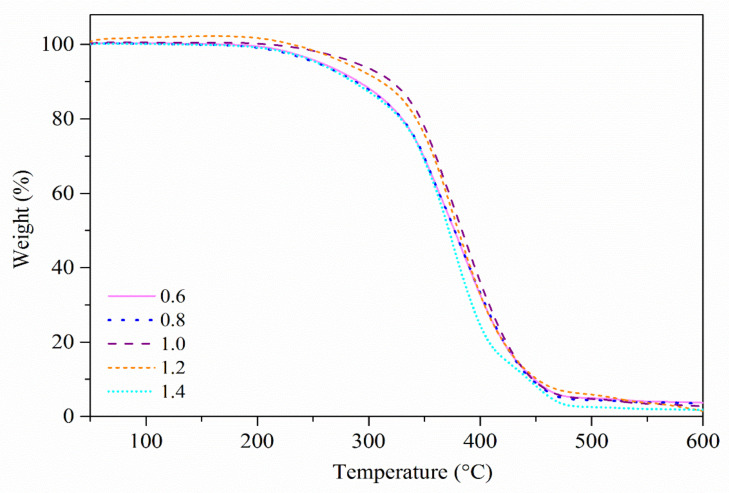
Thermogravimetric analysis (TGA) curves of EJO/MHPA with various ratios of anhydride to oxirane.

**Figure 7:**
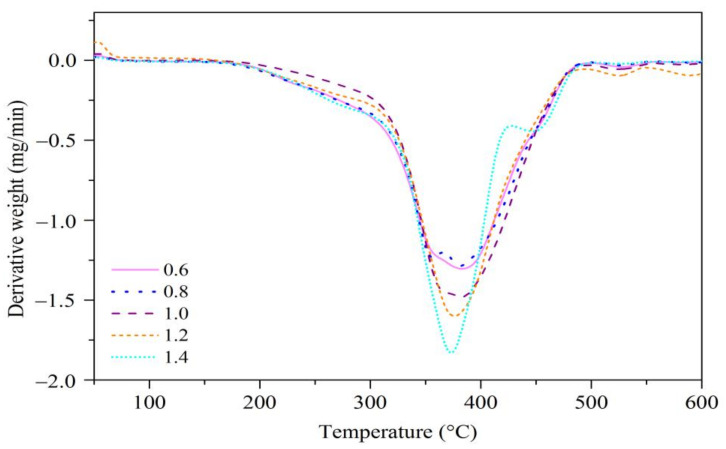
First-derivative weight curves (DTG) of EJO/MHPA polymers with various molar ratios of anhydride to oxirane.

Thermogravimetric analysis (TGA) is a commonly used technique to study the thermal properties of a polymer based on the degradation temperature, residuals, and other information obtained from the analysis [33]. The TGA curves of the EJO/MHPA polymers after curing at various ratios are shown in Figure 6, whereas their first-derivative weight curves (DTG) are depicted in Figure 7.

TGA data were analyzed based on its initial onset temperature (*T_i_*), decomposition temperature (*T_d_*) at different weight losses (wt), which were 5, 25, and 50% (T_5%_, T_25%_, and T_50%_), and weight residue % at 600 °C. All of the EJO/MHPA polymers had a single-step degradation pattern, and there was no evidence of unreacted chemicals that would volatilize before reaching the temperature of polymer degradation [34]. The temperatures corresponding to the T_5%_ for all samples were recorded above 240 °C, indicating good thermal stability. Overall, the analysis of various molar ratios showed that a ratio of 1.0 had the best thermal stability. This can be explained by the optimum crosslinking of the polymer network for the EJO/MHPA polymer with a molar ratio of 1.0 compared to other ratios, whereby it required more heat to break the bond. The peak temperature of mass losses significantly increased from a ratio of 0.6 to 1.0 and decreased for ratios of 1.2 and 1.4, which could be observed from the DTG thermograms. Details on the thermal properties of the EJO/MHPA polymers are shown in Table 1. From this, it can be concluded that the molar ratio of 1.0 of the EJO/MHPA polymer has better thermal stability than other ratios.

**Figure 8:**
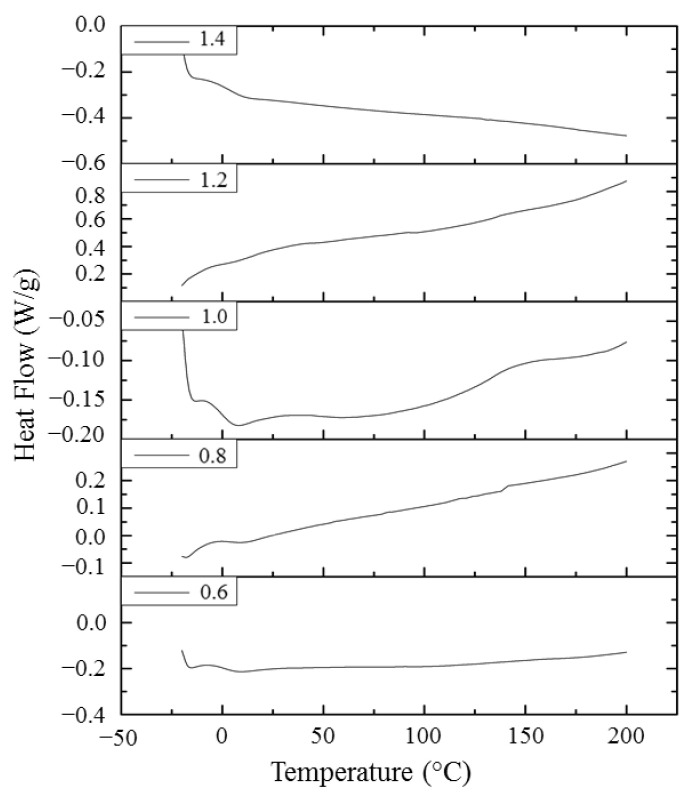
DSC curves of EJO/MHPA polymers with various ratios of anhydride to oxirane.

The glass transition temperature (*T_g_*) is considered as a fundamental polymer characteristic related to polymer properties and processing. In general, polymers with high crosslinking density have higher *T_g_*; however, the composition in the polymer within the cross-linked structure also plays an important role in the *T_g_* behavior. As is well-known, DSC is a widely used instrument to characterize *T_g_* of polymer materials [35]. Figure 8 and Table 2 summarize the thermal properties of EJO/MHPA polymers found in DSC curves, such as glass transition (*T_g_*) and enthalpy of transition (ΔH). All synthesized EJO/MHPA polymers showed only glass transition temperature values, but there was no presence of melting or crystallization peaks in DSC curves, which suggests that these EJO/MHPA polymers were amorphous. The curves also did not show an exothermic transition above 100 °C, which confirm the complete cross-linking between the epoxy group and anhydride during the curing cycle. The various molar ratios of EJO/MHPA polymers showed different values possibly due to the chain flexibility. For the amorphous polymer, *T_g_* values can serve as the shape transition temperature (*T_g_*). DSC data suggested that the *T_g1_* of the EJO/MPHA polymers were T_g1_ between 7.8 and 11.3 °C. The second glass transition was above 100 °C, where they could recover back to their original shape. However, the peak of *T_g2_* was too small to detect, and the ratios of 1.2, 1.0, and 0.8 had higher temperatures of *T_g_* peak, as can be seen in Figure 8. SMPs are divided based on their switch type into either *T_g_*-type SMPs with an amorphous phase or *T_m_*-type SMPs with a crystalline phase. As for the *T_g_*- type SMPs, a large rubber modulus can usually be maintained above the T_g_. *T_g_*-type SMPs exhibit relatively slow shape recovery compared with *T_m_*- or *T_g_*-type SMPs due to their broader glass transition interval, which hinders their application where immediate shape recovery is required [36].

### 3.4. Shape Memory-Recovery Behaviours

A study on the shape memory recovery behaviors of the EJO/MHPA polymers was performed. The primary shape of EJO/MHPA was in a linear rectangular shape. Then, the polymer was heated at 150 °C and cooled at room temperature. All of the EJO/MHPA polymers with various molar ratios showed good recovery ability and reached their original shape quickly within 30 s when they were heated above their glass transition temperature. The fundamental mechanism behind the shape memory behavior is the activation and freezing of the motion of the SMP chains above and below the *T_g_* [36], and the mechanism can be explained as follows: Firstly, the macroscopic deformation is translated to conformational change of the polymer molecular segments. Above *T_g_*, the polymer is in a rubbery state and can deform easily. Then, the temporary shape is fixed with the internal stress of the polymer network by cooling. When the EJO/MHPA is reheated above the *T_g_*, the heating causes rearrangement of molecular segments of the polymer network. Then the microscopic deformation is released, and the original shape of the EJO/MHPA polymers is recovered [5]. Shape-memory behavior can be exploited and demonstrated in various polymer systems significantly different in molecular structure and morphology. The common conventional SMP systems include cross-linked ethylene vinyl acetate copolymer, styrene-based polymers, acrylate-based polymers, polynorbornene, cross-linked polycyclooctene, epoxy-based polymers, thio-ene-based polymers, segmented polyurethane, and segmented PU ionomers. The intrinsic mechanism for shape memory behavior in thermal responsive SMPs is the reversible freezing and activation of polymeric chain motion in the switching segments below and above the transition temperature (T_trans_). The net-points of an SMP network, which maintain its dimensional stability, could be either covalent or physically crosslinked [36,37].

**Figure 9:**
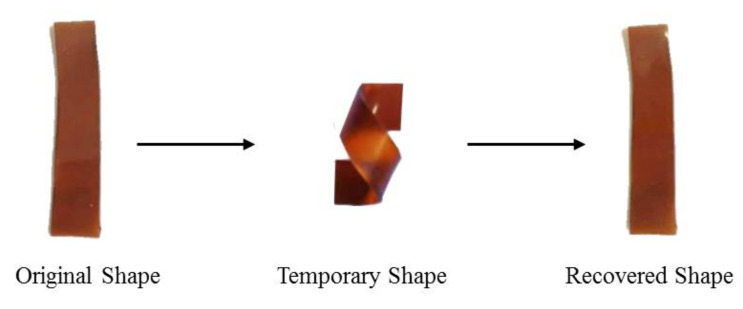
A demonstration of the shape memory recovery behaviors of EJO/MHPA polymers reported in this paper.

The shape memory effect (SME) is an extrinsic property of smart materials that allows them to restore or maintain their original shape even after being severely and quasiplastically distorted in the presence of the right stimuli [38]. According to previous research, one-way SME and two-way SME are two types of shape memory polymers with non-reversible and reversible shape-shifting properties, respectively [37]. One-way SME can retain a temporary shape once the stimulus is terminated. For two-way SME, the temporary shapes can be recovered to the initial shape when the stimulus is terminated. Reversible behavior occurs during heating and cooling in the presence or absence of external stress. In this study, EJO/MHPA polymer agreed with the two-way SME theoretically reported [39]. Figure 9 shows the demonstration of the shape-memory behaviors of EJO/MHPA polymers at molar ratio of 1.0. Moreover, the composition of epoxidized oil and anhydride curing agent play important roles in shape recovery due to crosslink density and chain flexibility.

## 4. Conclusions

We presented the preparation and characterization of bio-based epoxy material from jatropha oil for use in a shape memory polymer application. FTIR analysis showed the transition from jatropha oil to epoxidized oil through the disappearance of unsaturation peaks and formation of new epoxy peaks. The EJO was used to prepare bio-based polymers by curing this functionalized oil with MHPA acid anhydride and catalyzed with TEAB. The reaction produced slightly transparent and brown color polymers. The thermal properties of the EJO/MHPA polymers of different feed molar ratios of anhydride to oxirane were significantly different, perhaps due to the cross-link density and chain flexibility. The glass transition temperature of the EJO/MHPA polymers was between 7.8 and 11.3 °C. The shape memory polymer showed that the ratio of the anhydride to oxirane of 1.0 had better thermal stability compared to other ratios. Conclusively, the EJO/MHPA polymers showed good shape memory recovery behaviors. It is expected that this work could provide insight and an alternative approach for the production of shape memory polymers from current technology.

## Figures and Tables

**Table 1:** Thermal properties of EJO/MHPA polymers from TGA and DTG.

EJO/MHPA Ratio	*T_i_* (°C)	*T_d_* at Different wt. Losses (°C)			*T_d_*_max_ (°C)	Wt. Residue at 600 °C (%)
		*T*_5%_	*T*_25%_	*T*_50%_		
0.6	163	252	334	369	382	3.69
0.8	147	249	335	371	381	3.44
1.0	168	283	347	375	383	2.64
1.2	150	273	343	372	376	1.00
1.4	151	254	338	368	373	1.75

**Table 2:** Thermal properties of EJO/MHPA polymers found in DSC analysis.

EJO/MHPA Ratio	*T_g_*_1_ (°C)	*T_g_*_2_ (°C)	Δ*H* (J/g)
0.6	9.8	135	−3.76
0.8	10.8	140	−1.07
1.0	9.6	144	−2.86
1.2	7.8		−0.76
1.4	11.3		−2.00

## Data Availability

The data presented in this study are available on request from the corresponding author.
